# Effects of a pre-and post-workout protein-carbohydrate supplement in trained crossfit individuals

**DOI:** 10.1186/2193-1801-3-369

**Published:** 2014-07-21

**Authors:** Jordan J Outlaw, Colin D Wilborn, Abbie E Smith-Ryan, Sara E Hayward, Stacie L Urbina, Lem W Taylor, Cliffa A Foster

**Affiliations:** Human Performance Lab, Exercise and Sport Science Department, University of Mary Hardin-Baylor, 900 College Street, Belton, TX 76513 USA; Department of Exercise and Sport Science, University of North Carolina Chapel Hill, 209 Fetzer Hall, CB# 8700, Chapel Hill, NC 27599 USA

**Keywords:** CrossFit, Pre-workout, Post-workout, Supplement, Pomegranate

## Abstract

**Purpose:**

The purpose was to assess effects of a pre- and a post-workout protein-carbohydrate supplement on CrossFit-specific performance and body composition.

**Methods:**

In an open label randomized study, 13 male and 16 female trained Crossfit participants (mean ± SD; age: 31.87 ± 7.61 yrs, weight: 78.68 ± 16.45 kg, percent body fat: 21.97 ± 9.02) were assessed at 0 and 6 weeks for body composition, VO_2_max, Wingate peak (WPP) and mean power (WMP), in addition to sport-specific workouts (WOD1: 500 m row, 40 wall balls, 30 push-ups, 20 box jumps, 10 thrusters for time; WOD2: 15 minutes to complete an 800 m run "buy in", followed by as many rounds as possible (AMRAP) of 5 burpees, 10 Kettlebell swings, 15 air squats). The supplement (SUP) group consisted of 19 g of a pre-workout drink (extracts of pomegranate, tart cherry, green and black tea) taken 30 minutes before and a post-workout protein (females: 20 g; males: 40 g) and carbohydrate (females: 40 g; males: 80 g) supplement consumed immediately after each workout. The control (CTL) group consumed only water one hour before or after workouts. Participants completed three (minimum) varied workouts per week at a CrossFit gym as typical to habitual training throughout the six week study. Data were analyzed by repeated measures ANOVA (*p* <0 .05), 95% Confidence Intervals, and Magnitude Inferences.

**Results:**

There were no time × group interactions for body composition, WMP, or WOD1 based on ANOVA statistics. VO_2MAX_, WPP, and WOD2 results revealed that the pre/post supplements were likely beneficial after 95% Confidence Intervals and Magnitude Inferences analysis.

**Conclusion:**

The combination of proprietary supplements taken for 6 weeks may provide benefits during certain sport-specific performance in trained CrossFit athletes but not others.

## Introduction

Pomegranate extract, tart cherry extract, and green tea extract (caffeine plus catechins) have health benefits related to improved antioxidant function, improved body composition, and antiatherosclerotic properties (Guo et al. 2008; Howatson et al. 2010; Wang et al. 2010). Pomegranate, containing nitrates and polyphenolics molecules (Sumner et al. 2005), has been used by researchers to alter inflammation, interleukin-6, and the activity of antioxidants (Guo et al. 2003). In one such study, researchers discovered that trained individuals had greater strength and less muscle soreness in the upper body (elbow flexor) after performing eccentric exercise when taking a pomegranate juice supplement compared to consumingthea placebo (Trombold et al. 2011). Further analysis showed that there were no differences between consuming pomegranate juice and consuming a placebo on either lower body strength or muscle soreness (knee flexors). In an earlier study conducted by Trombold and associations, strength was greater at 48 and 72 hours post eccentric exercise-induced muscle damage (elbow flexor) after nine days of pomegranate extract supplementation versus placebo (Trombold et al. 2010). Blood analysis did not reveal any difference between groups that could further explain the mechanism of action.

Other research suggests that pomegranate pure juice significantly reduced urinary free cortisol after one week of supplementation. Participants completed two 30-minute treadmill exercise sessions before and after seven days of consuming 500 ml/day of either pomegranate juice or water. Systolic and diastolic blood pressure were both reduced significantly after the supplementation period (Tsang et al. 2011).

Antioxidant-rich cherry extract has been linked by researchers to decreased inflammation and recovery from intense exercise. In various studies, cherry supplementation led to improved recovery from exercise (Bowtell et al. 2011), decreased pain while running (Kuehl et al. 2010), and decreased strength loss after damaging exercise (Connolly et al. 2006).

Polyphenols in green tea, similar to pomegranate or tart cherry extract, have been linked to antioxidant enhancement and may be responsible for the inhibited increase in creatine kinase and xanthine oxidase associated with exercise (Ichinnose et al. 2011). Green tea extract also decreases respiratory exchange ratio during aerobic exercise after seven days of supplementation (Panza et al. 2008). Jowko and associates gave untrained males a green tea extract supplement for four weeks and measured markers of exercise-induced oxidative damage. After four weeks of strength training and supplementation, an upper and lower body muscular endurance test was performed. The supplement group had increased plasma total polyphenols, antioxidant status, and decreased creatine kinase activity 24-hours post-exercise (Jowko et al. 2011). In addition, green tea has known anti-obesity effects (Wolfram et al. 2006).

Furthermore, protein and carbohydrate after a workout is known to increase lean body mass and replenish glycogen stores in trained individuals (Borsheim et al. 2004a; Kerksick et al. 2008; Campbell et al. 2007; Tremblay et al. 1994; Tarnopolsky et al. 1997). Carbohydrate post-exercise (100 g) has been shown to decrease muscle protein breakdown compared to consuming a placebo (Borsheim et al. 2004b) and a small dose of protein (10 g whey protein) plus carbohydrate (21 g) increases synthesis significantly (Tang et al. 2007). According to the research, high-intensity exercise, similar to that performed during a CrossFit workout, combined with protein consumption leads to positive net protein balance (Hulmi et al. 2010). The increased muscle protein synthesis and inhibited protein breakdown leads to increase fat-free body mass. Ivy’s research has demonstrated the increase in glycogen resynthesis when protein and carbohydrates are combined and consumed immediately post-workout (Ivy et al. 2002) as well as the increase in muscle protein synthesis and muscle tissue repair (Ivy 2001). Combining these benefits may have a positive impact on an athlete’s performance and body composition although research is lacking in regards to athletes participating in high-intensity training such as CrossFit. The purpose of the current study was to investigate changes in body composition, sport-specific performance, and aerobic and anaerobic capacity after six weeks of pre-workout and post-workout supplementation combined with varied, high-intensity exercise in trained CrossFit individuals.

## Methods

### Participants

Twenty-nine participants (n = 29, males = 13, females = 16; mean ± standard deviation; age: 31.87 ± 7.61 years; weight: 78.68 ± 16.45 kg;% body fat: 21.97 ± 9.02%) completed the six week randomized, open label study. All participants had been participating in at least three CrossFit workouts per week for at least six months. Participants were required to be apparently healthy (based on completed medical history questionnaires) and had not used dietary supplements that could influence physical performance or body composition in the past three months. Testing procedures were explained to all volunteers at the familiarization session. Participants completed medical and physical activity history questionnaires and read and signed Informed Consent statements (approved by the University IRB) prior to the start of the study.

### Familiarization

Before beginning baseline testing, participants were required to attend a familiarization session. At the familiarization session, in addition to completing paperwork and signing the Informed Consent statements, participants completed a practice VO_2MAX_ graded exercise test and Wingate power test to become familiar with testing protocols.

### Testing

After attending the familiarization session, participants completed two Workouts of the Day (WOD1 and WOD2) at a CrossFit gym. WOD1 required the participant to complete a 500 m row, 40 wall balls, 30 push-ups, 20 box jumps, and 10 thrusters as quickly as possible. The time to completion was recorded in seconds. After a 20 minute rest, participants then completed WOD2 which consisted of an 800 m run "buy in" (only completed once) followed by as many rounds as possible (AMRAP) of 5 burpees, 10 Kettlebell swings, and 15 air squats within 15 minutes. At the end of WOD2, the number of completed repetitions was recorded. Participants again reported to the Human Performance Lab (HPL) to complete baseline (T1) testing (anthropometric, aerobic, and anaerobic) within 48 hours of completing WOD1 and WOD2. Participants repeated testing measures (T2; VO_2MAX_, Wingate, and body composition) 24 to 48 hours after completing both WODs at the end of the six-week supplementation period.

Height and weight were measured at T1 and T2 using the SECA 242 measuring instrument (242, SECA, Hanover, MD) and the TANITA Body Composition Analyzer (Model TBF-310, TANITA, Arlington Heights, IL), respectively. A Dual-Energy X-ray Absorptiometry scan (DEXA) was then completed using the Hologic Discovery (Hologic, Inc., Bedford, MA). Body fat percentage (%BF), fat mass (FM), and lean body mass (LBM) were recorded from the DEXA scan printout.

A maximal oxygen consumption running treadmill test (VO_2MAX_) was completed during the familiarization session and at T1 and T2 to determine aerobic capacity. Participants were fitted with a mouth piece and head apparatus then instructed to walk or run for as long as possible. The Bruce protocol was utilized, which provides the participant with a three minute warm-up followed by successive three minute stages that provide an increase in speed and incline. All expired air traveled through the mouth piece and into the TrueOne® 2400 (ParvoMedics, Sandy, UT) metabolic cart to be analyzed by the computer. The VO_2MAX_ value in mL/kg/min was recorded after the completion of the test.

After 15 minutes of rest, the Wingate power test was used to analyze anaerobic capacity. Utilizing the Excalibur Sport V2 (LODE, Groningen, The Netherlands) bicycle, a Wingate protocol was followed. The participant was instructed to maintain RPMs at 60–80 throughout the two minute warm-up. Immediately before the 30-second test, the participant was told to pedal as hard and as fast as possible while remaining seated. For 30 seconds, the load placed on the fly-wheel remained constant [(0.7 N·kg^-1^ body mass) × (participant’s body mass (kg)]. Peak power (WPP) and mean power (WMP) were recorded in Watts.

All participants completed a four day (4-d) diet log (two week days, two weekend days) prior to T1 and T2. In addition, a one day (1-d) diet log was completed on the last workout day of each week throughout the supplementation period. Participants were asked to report all food and beverage intake (excluding water) by writing the name of the food, the method of preparation, and the quantity consumed. Diet logs were analyzed using the Food Processor (esha Research, Salem, OR) computer software and a four day average (T1 and T2) for total calories, protein, carbohydrate, and fat consumed were recorded.

### Supplementation and training

Participants were matched based on sex and number of days they participated in CrossFit per week and then randomly assigned to either the supplement group (SUP) or the control group (CTL). SUP participants received a pre-workout supplement (19 g, Pursuit Rx Pre-Workout, Dymatize Nutrition, Dallas, TX) that was to be taken thirty minutes before each CrossFit workout. The pre-workout supplement contained Pomegranate Fruit Extract (NITRO_2_GRANIT™), Tart Cherry Extract, Beet Root Extract, Green Tea Extract (AssuriTEA™, Kemin, Dubuque IA), and Black Tea Extract (InnovaTEA® , Kemin, Dubuque IA). Participants also were given a whey protein and carbohydrate supplement (Pursuit Rx Recovery Blend, Dymatize Nutrition, Dallas TX) to mix with water and consume immediately after each CrossFit workout. Females were given two scoops (1 serving; 20 g protein; 40 g carbohydrate) of the supplement to combine with 8–10 oz of water and males were given four scoops (2 servings; 40 g protein; 80 g carbohydrate) to combine with 16–20 oz of water. The control group was instructed to refrain from taking any performance oriented dietary supplements over the course of the study and required to consume only water one hour before and one hour after each CrossFit workout.

After every CrossFit workout, participants completed a perception of mood survey, a rate of perceived exertion (RPE) questionnaire, and delayed-onset muscle soreness (DOMS) survey. At the end of each week, the SUP group completed a supplement follow-up questionnaire to ensure compliance and monitor for any side-effects from the supplements.

At baseline testing, participants reported the average number of CrossFit workouts they routinely participated in (3–5 workouts per week) and were told to maintain this level of activity throughout the study. The WODs performed at the CrossFit gym were not the same as WOD1 or WOD2 performed at T1 and T2.

### Data analysis

All performance variables were analyzed by a group (SUP vs CTL) × time (T1 vs T2) repeated measures ANOVA. The alpha level was set at 0.05. Confidence Intervals (95%) and magnitude inferences were used to analyze performance variables and body composition measurements. These progressive statistics were used to further investigate meaningfulness and practical importance, as outlined by Hopkins, Marshall, Batterham, and Hanin (Hopkins et al. 2009). For determining the degree of beneficial effects, the following ranges were used: <0.5%—most unlikely, almost certainly not; 0.5-5%—very unlikely; 5-25%—unlikely, probably not; 25-75%—possibly; 75-95%—likely, probably; 95–99.5%—very likely; >99.5%—most likely, almost certainly (Hopkins et al. 2009).

## Results

All participants completed the study and compliance for nutritional intervention and weekly workouts was assured through post-workout surveys and diet logs. Analysis of the dietary journals suggested that daily intake of protein as well as carbohydrate intake significantly increased from T1 to T2 for the SUP group compared to CTL (Protein: +21.64 g vs -9.50 g, p = 0.023; Carbohydrate: +23.34 g vs -46.19 g, p = 0.016) while fat (g) and total calories did not change. The statistical analysis revealed that there were no group × time interactions for any of the measured variables. 95% Confidence Intervals and magnitude inferences revealed that the supplement had no effect on measures of body composition (% BF: p = 0.70, FM: p = 0.80, LM: p = 0.41). The supplement group recorded an increase of FFM of 1.67%, which is statistically insignificant, but may be of interest to highly trained individuals as the ability to make improvements decreases as performance and training status increase.

**Figure 1:**
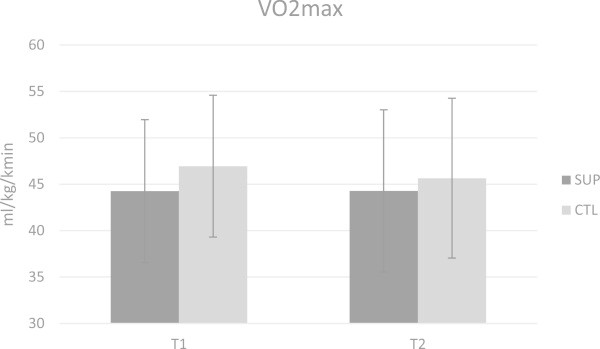
**Changes in VO**_**2MAX**_ **were not significant between groups according to ANOVA analysis.** There were likely beneficial effects based on magnitude inferences suggesting that the supplement group likely benefited from the supplements compared to the control group.

**Figure 2:**
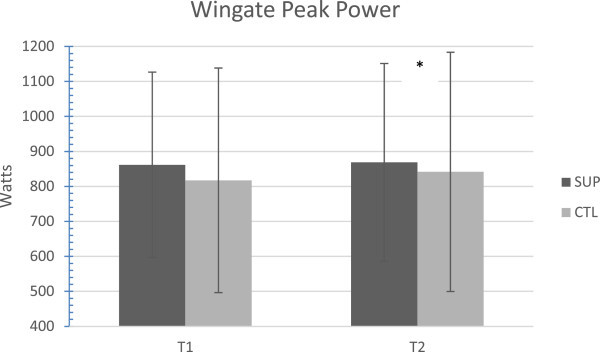
**Magnitude inferences revealed that the supplements had a likely beneficial effect on the changes that occurred in the SUP group.** There was a significant difference between groups (confidence intervals). * represents *p* ≤ 0.05. SUP: supplement.

**Figure 3:**
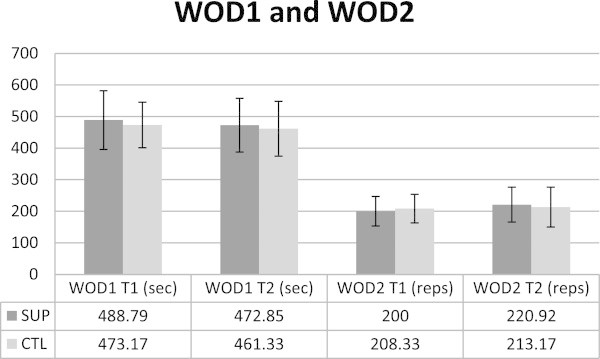
**WOD1, measured in time, improved for both groups and there was no significant difference between SUP and CTL.** WOD2 performance likely benefited from the supplement for SUP based on magnitude inferences. There was no significant difference between groups. WOD1: workout of the day 1, SUP: supplement, CTL: control, WOD2: workout of the day 2.

The pre-workout supplement plus the post-workout protein had likely beneficial effects on VO_2MAX_ (78.16%) during recovery period and Wingate peak power (73.40%) over the duration of the intervention timeframe based on statistical analysis (magnitude inferences and 95% confidence intervals). VO_2MAX_ was maintained from T1 to T2 for SUP whereas VO_2MAX_ decreased from T1 to T2 (Figure 1). Wingate peak power changes were also significant based on 95% Confidence Intervals. The performance in WOD1 and WOD2 were potentially meaningful with mean performance improvements in time in WOD1 of -38.79 sec (SUP; 5.85%) vs -8.62 sec (CTL; 2.39%) and +16.79 reps (SUP: 10.01%) vs +6.31 reps (CTL; 2.41%) in AMRAP in WOD2. Although the time to completion was not determined to be significantly different for WOD1 T1 to T2, changes in WOD2 performance were likely beneficial (84.95%) for the supplement group according to magnitude inferences (SUP: +9.59 ± 10.91 W; CTL: -0.88 ± 18.25 W). Wingate mean power values can be seen in Figure 2, WOD1 and WOD 2 data is presented in Figure 3.

## Discussion

After six weeks of on-going CrossFit training and pre- and post -workout supplementation, the supplement group had likely beneficial changes in measures of cardiorespiratory fitness, anaerobic power, and sport-specific performance. The pre-workout contains pomegranate fruit extract that is standardized for polyphenols and nitrates. Based on other research involving nitrates and performance, dietary nitrate can decrease the demand for oxygen during aerobic exercise, allowing the exerciser to become more efficient (Larsen et al. 2007). Furthermore, dietary nitrate given for two days before testing lead to a small decrease in VO_2MAX_ but allowed the participants to exercise for a longer duration (Larsen et al. 2010). When a nitrate supplement (beetroot juice) was given to eight adult men (19–39 years), time to exhaustion during severe exercise was improved, oxygen uptake at the onset of moderate exercise was reduced, and systolic blood pressure was reduced (Bailey et al. 2009). These adaptations in oxygen consumption and efficiency would explain the maintenance on VO_2MAX_ in the SUP group and the decrease in the CTL group. It may be of interest for future researchers to investigate the effects of chronic dietary nitrates taken by trained CrossFit participants.

Improved recovery after muscle damaging exercise was seen by Bowtell and colleagues when participants were given either cherry juice concentrate or a placebo for seven days before inducing muscle damage of the knee extensors (Bowtell et al. 2011). Maximum voluntary contraction force recovered more quickly in the supplement group than the placebo. The participants drinking cherry juice also had a lower absolute and percentage increase in protein carbonyls, a marker of oxidative stress resulting in the breakdown of amino acids (Dalle-Donne et al. 2003). These results suggest that cherry juice can counteract the damaging effects of intense exercise. If applied to the current study, it seems possible that the pre-workout supplement assisted the participants in recovering from the previous exercise bout and this might be reflected in the better recovery of VO_2_max and improvement of WPP from T1 to T2. It seems logical that if one can recover more-quickly from exercise, then he or she will be more prepared to handle the physical stress of the subsequent workout.

Cherry juice supplementation (7-d) has also been shown to decrease the pain associated with running long distances although it is unclear whether this benefit could transfer over to resistance training but is an area for future research (Kuehl et al. 2010). Considering the nature of the daily workouts in this sport, it is clear that the study participants did not train specifically for the lab tests. Wingate peak power, which was analyzed as 73.4% likely beneficial (magnitude inferences), suggests that the supplement had positive effects on the anaerobic system when combined with workouts that are varied in nature compared to the control.

Green tea extract may have played a similar role as that of pomegranate and tart cherry extract in terms of enhanced antioxidant activity although plasma analysis would be needed to further assess this variable. Additionally, caffeine can have positive effects on alertness, focus, and concentration and the pre-workout used in this study provided 160 mg derived from the tea ingredients. Caffeine and caffeine-containing supplements are often used by athletes to enhance performance by delaying fatigue (Mohr et al. 2010) and decreasing reliance on muscle glycogen stores (Goldstein et al. 2010). Green tea may have had an effect on plasma free fatty acid levels, which have been reported to increase with green tea consumption (Murase et al. 2006), but fatty acid levels were not measured during the present study. In the future, researchers should investigate plasma free fatty acid levels and measure glycogen storage while consuming the products used in the current study.

In a study investigating the effects of caffeine on high-intensity interval exercise (repeated Wingates), caffeine had no effect on the first two Wingates and had a negative effect on the last two compared to a placebo (Greer et al. 1998). Further research should be done to determine if the caffeine in the current pre-workout supplement (160 mg) played a role in the improved WOD2 performance.

While the pre-workout supplement was not consumed before T2 testing, the enhanced glycogen stores from the post-workout supplement perhaps allowed the SUP group to generate more power during the Wingate test. This, of course, is speculative as muscle glycogen content was not measured during this study. The combination of carbohydrate and protein ingested after a workout session has shown to improve protein synthesis rates as well as increase glycogen re-synthesis (Campbell et al. 2007) and future research should focus on the benefits of glycogen loading for CrossFit athletes and how to maximize the benefits through nutrition. Further research should also be done on the post-workout protein-carbohydrate supplement to determine the effects it has on glycogen stores during CrossFit training.

Additionally, consuming protein immediately post-exercise has been shown to increase lean body mass and create an overall anabolic environment when combined with resistance training (Willoughby et al. 2007). The lack of significant body composition changes could be attributed to the calorie count of the post-workout protein and carbohydrate supplement as well as the overall eating habits of the participants. Male participants consumed four scoops (480 calories) and female participants consumed two scoops (240 calories) immediately after exercise. Factoring in the dietary changes of both groups, one potential issue could be differences in protein and carbohydrate intake. Protein and carbohydrate intake increased for SUP and decreased for CTL. This difference could be due to the fact that the CTL group was asked to not eat one hour before or after workouts, leading to a decrease in overall protein and carbohydrate consumption from T1 to T2. Future research should focus on energy needs of athletes participating in high-intensity functional resistance training similar to CrossFit to determine the most beneficial combination of protein and carbohydrate post-exercise.

One specific limitation to this study is the lack of a placebo-controlled group, although it is common for investigators to utilize an open-label design. With this is mind, there is the need for future research on the utilized supplements with both a supplement and placebo group.

## Conclusion

The proprietary blend pre-workout supplement used in the present study may be beneficial for increasing power (74.40% likely beneficial) and maintaining VO_2MAX_ (78.16% likely beneficial) during CrossFit-type training. Further research should be conducted to determine what other sport-related benefits may be achieved with the proprietary blend supplements (recovery during extended competition, recovery from multiple WODs, etc.).
